# Supplementary material for: CellWalker integrates single-cell and bulk data to resolve regulatory elements across cell types in complex tissues
Source: Genome Biol. 2021 Feb 14;22:61. doi: 10.1186/s13059-021-02279-1 (PMC7883575; doi:10.1186/s13059-021-02279-1)
Supplement: Supplementary file 1 — Additional file 1: Fig. S1. Flowchart of CellWalker Pipeline. Fig. S2. Additional Simulation Results. Fig. S3. Runtime Analysis. Fig. S4. CellWalker Performance on SNARE-seq Data. Fig. S5. nEN Progression. Fig. S6. Cell Type-Specific Regulatory Elements. [file 13059_2021_2279_MOESM1_ESM.pdf]

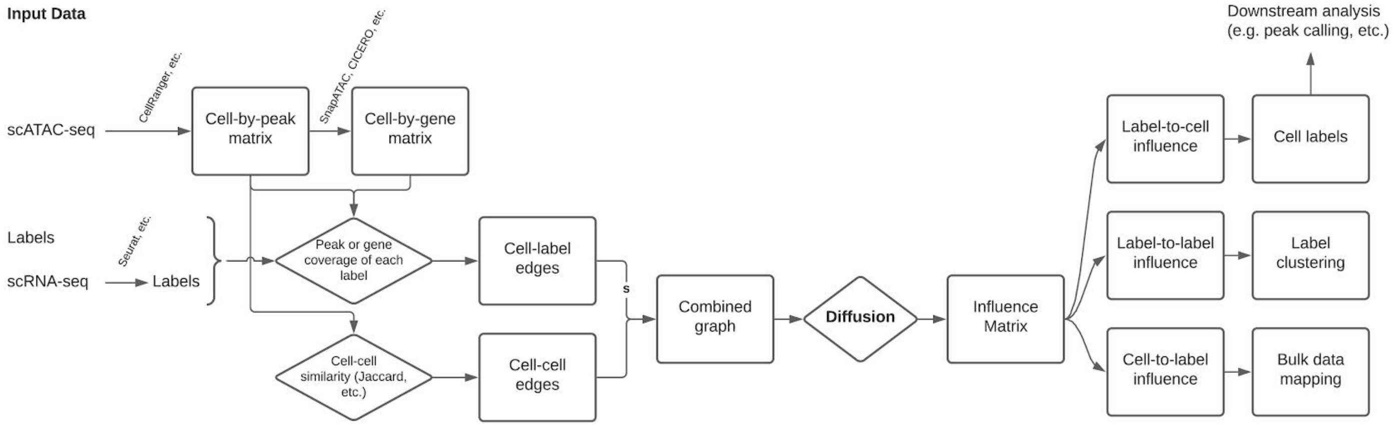

Fig. S1**. Flowchart of CellWalker Pipeline.** CellWalker takes as input scATAC-seq data and labeling information, either directly in the form of marker genes, or by processing scRNA-seq data to generate labels. scATAC-seq data can optionally be converted into a cell-by-gene matrix using software such as SnapATAC, ArchR, or CICERO. The cell-by-peak or cell-by-gene matrix is combined with the labels to generate cell-label edges. Cell-to-cell edges are directly computed from the cell-by-peak matrix. A full network is built using these two sets of edges and a label edge weight parameter *s*. A graph diffusion is computed on the combined network to generate an influence matrix which includes three portions used for downstream analysis: label-to-cell influence, label-to-label influence, and cell-to-label influence. These are used for cell labeling, label clustering, and bulk data mapping, respectively. Cell labels can then be used further for peak calling, transcription factor binding, and other analyses provided by software such as SnapATAC, cisTopic, or ArchR.

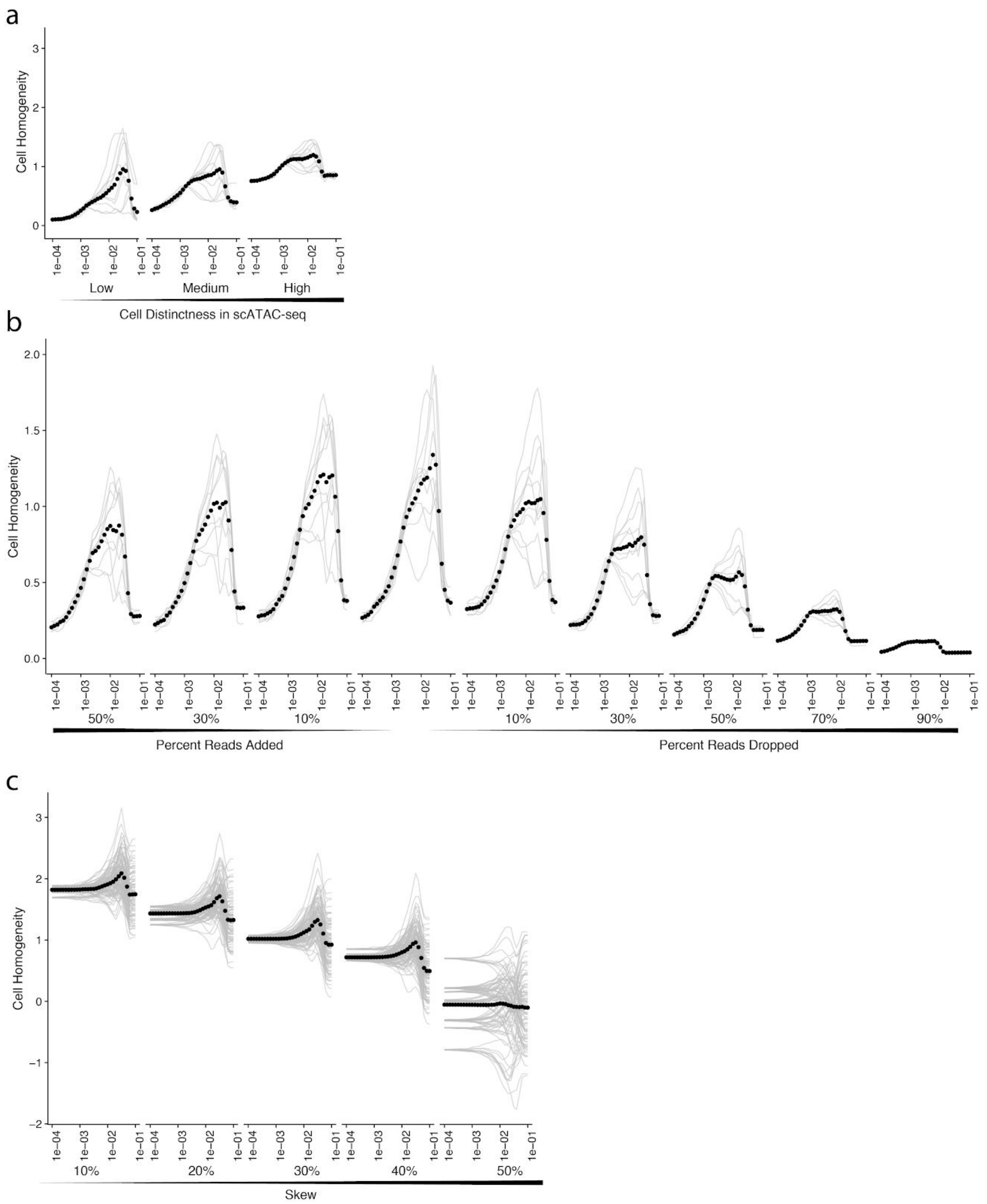

Fig. S2**. Additional Simulation Results. a.** As the within-type cell distinctness increases (x-axis), *de novo* cell homogeneity increases a large amount (first point on each curve), but optimal cell homogeneity increases only slightly, indicating that within-cell type similarity can be low to achieve high cell homogeneity. **b.** As simulated reads are randomly added or removed (x-axis), cell homogeneity decreases. Adding random reads (left) only slightly decreases cell homogeneity. Removing reads (right) has a larger effect; even as many as 50% of reads can be dropped and optimal performance is still higher than the highest de-novo cell homogeneity. **c.** An unlabeled population of cells is less and less skewed towards one cell type (x-axis), with the proportion of bins from the cell type adjusted between 10 and 50 percent. Until the unlabeled cell type is exactly equally sampled from the other two types, cell homogeneity (in this case computed as the ratio of influence between the unlabeled cells and the two sets of labeled cells) remains high. Each level of skew includes 10 repeated random relabelings of 10 randomly generated sets of skewed cells.

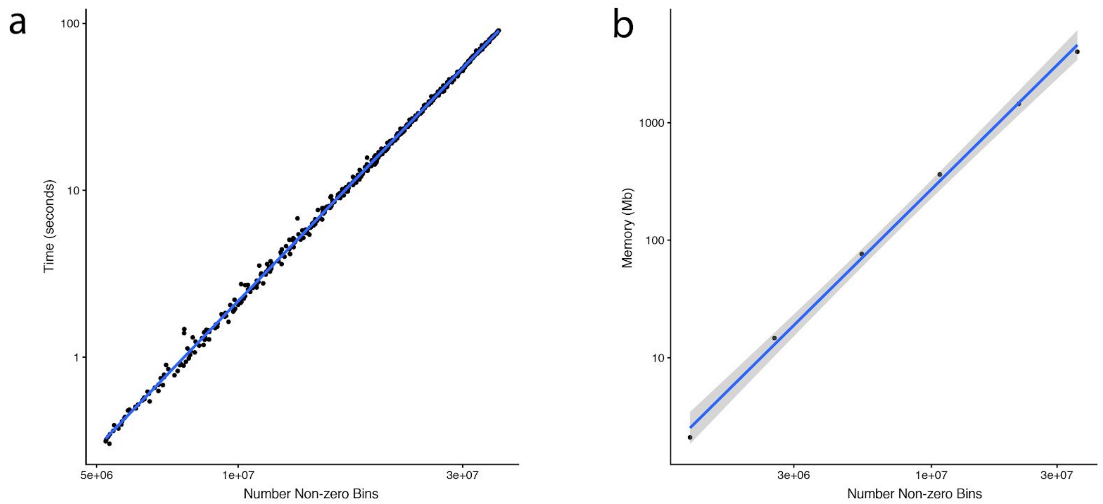

Fig. S3. **Runtime Analysis. a.** Running time for CellWalker (y-axis) versus the number of non-zero bins in the cell-by-bin matrix representing scATAC-seq data (x-axis). The blue line shows the best fit of a linear model with standard error shown in gray. In our data, cells had a median of around 6,000 non-zero entries. We can extrapolate that running on a single 2.2 GHz core, it would take about 8 minutes of clock time to run CellWalker on a set of 10,000 cells, 11.7 hours for 50,000 cells and ~80 hours for 100,000 cells. **b.** Memory usage of CellWalker versus the number of non-zero bins. The blue line shows the best fit of a linear model with standard error shown in gray. We can extrapolate that it would take about 20Gb of RAM to run CellWalker on a set of 10,000 cells, 788Gb for 50,000 cells, and >3Tb for 100,000 cells. Most scATAC-seq experiments sequence up to 10,000 cells, but looking ahead to larger experiments, 50,000 cells would still be feasible to analyze on a powerful server, while 100,000 cells would require a high-performance cluster.

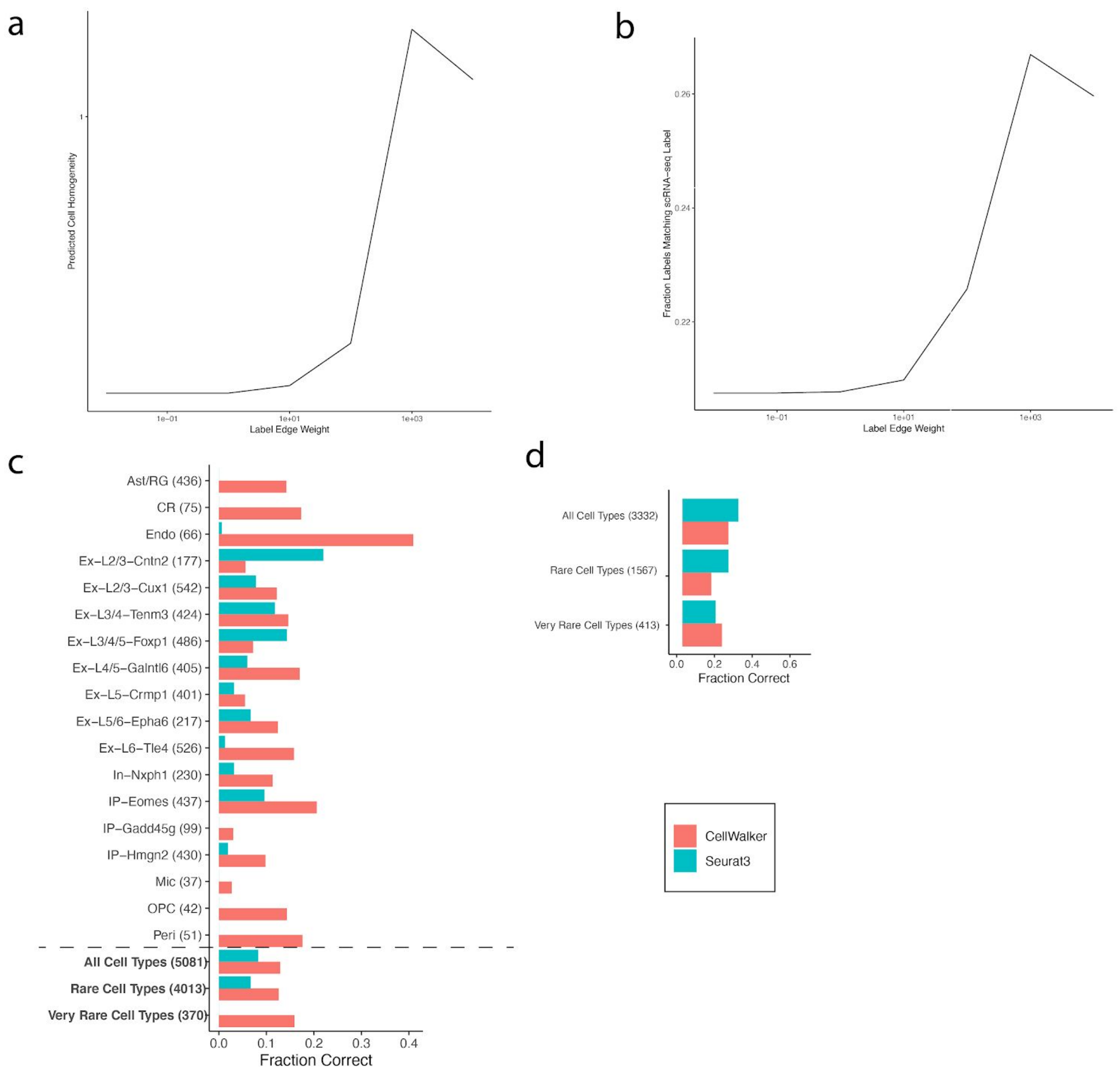

Fig. S4. **CellWalker Performance on SNARE-seq Data. a.** Cell homogeneity (y-axis) computed using the hidden scRNA-seq based cell labels as the median ratio of within-label versus out-of-label influence across possible settings of the label edge weight parameter (x-axis) **b.** Fraction of cells labeled the same as the hidden scRNA-seq based label (y-axis) across settings of the label edge weight parameter. The two measures peak near the same setting of the parameter. **c.** CellWalker correctly labels cells from the ATAC portion of developing mouse cortex SNARE-seq data (number of cells of each type in parenthesis) with no drop off for rare (max 500 cells) and very rare (max 100 cells) cell types. **d.** Fraction of cells labels in agreement with scRNA-seq clustering in a 10x Single Cell Multiome ATAC + Gene Exp chip for human healthy brain tissue. As there is no labeling data, scRNA clusters were generated using Seurat and assumed to be correct labels, giving Seurat a built-in advantage. This results in proportionally fewer cells labeled to rare cell types, though CellWalker still outperforms Seurat on those cells.

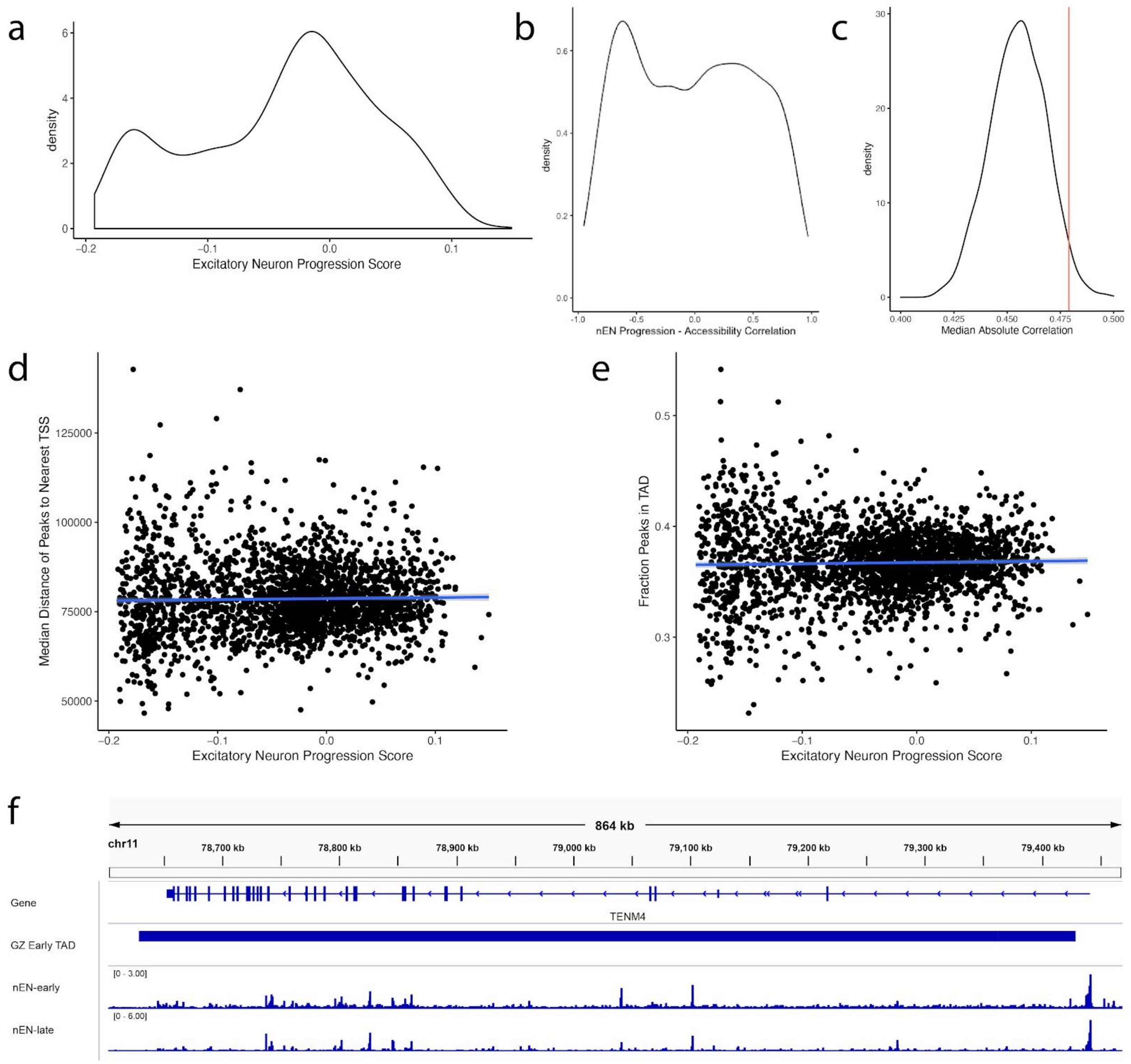

Fig. S5. **nEN Progression. a.** Distribution of excitatory neuron progression scores. **b**. The distribution of correlations between nEN progression scores and accessibility across GZ TADs **c.** The distribution of absolute correlation between TAD accessibility and nEN progression scores for random TADs as compared to true GZ TADs (red line, empirical p-value 0.021) **d.** Correlation between excitatory neuron progression score and the median distance from distal peaks to their nearest TSS. Each point is one cell, and the blue line is the best fit of a linear model. The two are not significantly correlated (Pearson's correlation coefficient 0.02, *p*-value=0.3) **e.** Correlation between excitatory neuron progression score and fraction of peaks in TADs. Each point is one cell, and the blue line is the best fit of a linear model. The two are not significantly correlated (Pearson's correlation coefficient 0.03, *p*-value=0.2). **f.** *TENM4* lies in a GZ Early TAD.

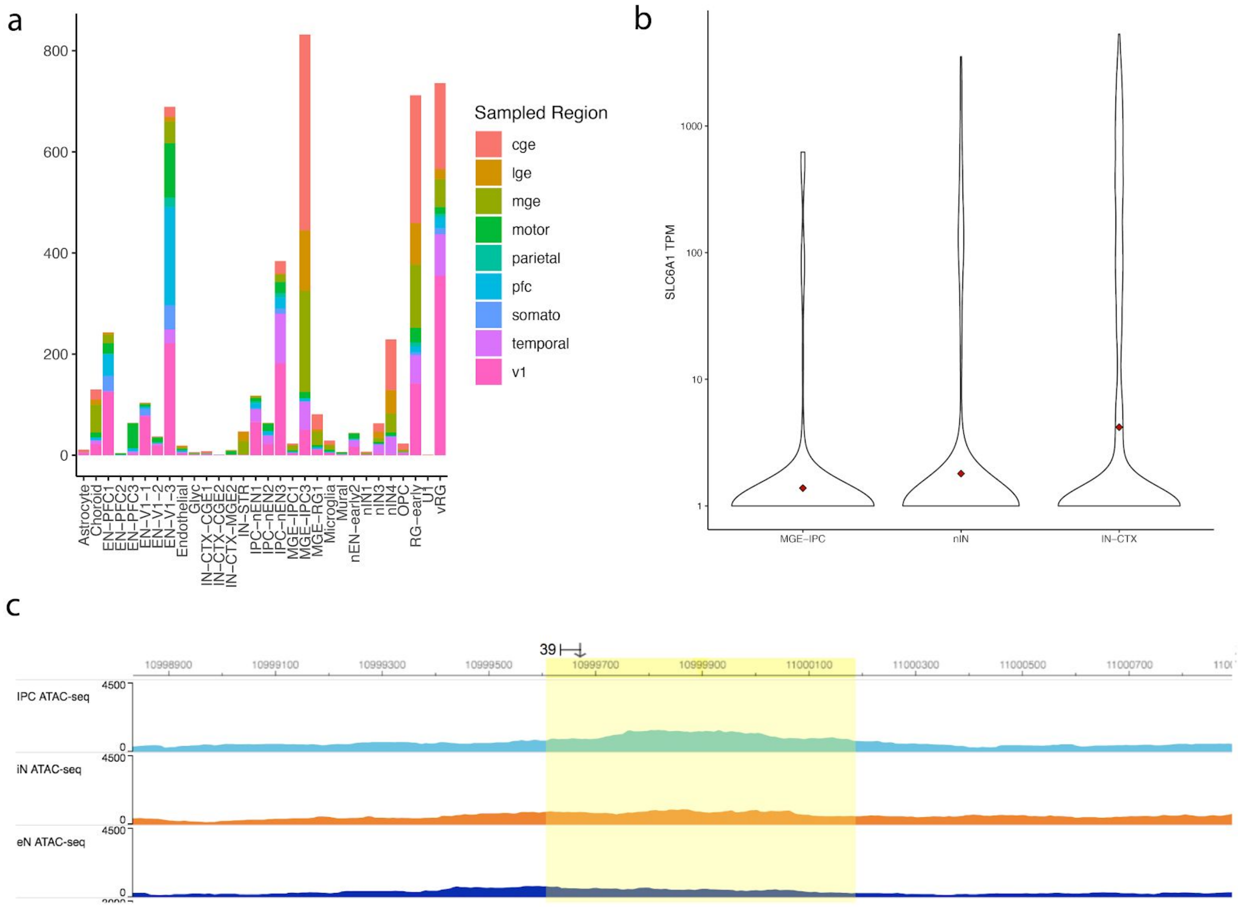

Fig. S6. **Cell Type-Specific Regulatory Elements. a.** The number of cell type-specific putative regulatory elements (pREs) that uniquely map to pREs from each microdissected region. **b.** TPM for *SLC6A1* for each cell in scRNA-seq data with mean shown in red. **c.** Peaks in bulk ATAC-seq data for FACS sorted cells in the *SLC6A1* enhancer (yellow highlighted region).
