# Supplementary material for: CellWalker integrates single-cell and bulk data to resolve regulatory elements across cell types in complex tissues
Source: Genome Biol. 2021 Feb 14;22:61. doi: 10.1186/s13059-021-02279-1 (PMC7883575; doi:10.1186/s13059-021-02279-1)
Supplement: Supplementary file 3 — Additional file 3. Review history. [file 13059_2021_2279_MOESM3_ESM.docx]

Review History

**First round of review**

**Reviewer 1**

**Were you able to assess all statistics in the manuscript, including the appropriateness of statistical tests used?**

No

**Were you able to directly test the methods?**

Yes

**Comments to author:**

In this manuscript, the authors developed a new approach to integrate the information from bulk assays with scATAC-seq to improve the annotation of cell identity and tissue- and development-specific regulatory elements. This is an interesting method with promising potentials. However, the clarity of the current manuscript needs to be dramatically improved. The omission of some key details made it very difficult to evaluate some important results and conclusions.

Major points:

1. The method section on how the algorithm work needs more details. I found the information that the authors provided in the GitHub are quite helpful. I'd suggested that the authors include those GitHub contents to this manuscript and provide a flowchart to better demonstrate the overall workflow of CellWalker.

2. The authors used the ATAC-seq signals around promoters and gene bodies to link the gene-expression based label information to cells. It seems that the distal enhancers, which are more important to tissue-specific expression, were not included when building these label-cell edges. Can the authors explain the rationale behind?

3. scATAC-seq in human telencephalon is one of the major data set the authors used to demonstrate the power of CellWalker. However, description of this data set is not clear. I can understand that the authors tried to avoid introducing overlapping contents with another manuscript which seems still under review. However, there should be enough information provided so that the readers do not have to read through another manuscript just for some fundamental information. For examples, How the cells for scATAC-seq were prepared in that study? How CellWalker improved the annotation of different cell populations, especially compared with other approaches?

4. In "Cell-type specific annotation of loci" section. Instead of directly jumping to the aggregated analysis of signals within TADs, what is the genome-wide accessibility change of individual ATAC-seq peak among different cell types annotated by CellWalker? It seems some pieces information were missing in this section. In addition, it is not accurate to call those cell-type specific TADs, since I did not find any data showing that the TAD boundaries changed among different cell types.

5. In "Cell-type specific annotation of enhancers" section. Again, due to the limited information on data sets description. It is difficult to judge the biological relevant of the bulk ATAC-seq and scATAC-seq data used in the analysis. In addition, I do not feel the author have enough information to call those ATAC-seq peaks enhancers. "Regulatory elements" is more accurate.

Minor points.

6. Line 121 "When edge weight is low, the output is similar to de- novo cell clustering using only scATAC-seq" one comma is missing between the two sentences.

7. Line 159 : only the cluster of scATAC-seq data is shown in Figure 3a, I failed to see " high agreement with cluster based on scRNA-data"

8. Line 202, pls show the distribution instead of only a p-value.

**Reviewer 2**

**Were you able to assess all statistics in the manuscript, including the appropriateness of statistical tests used?**

Yes: statistics is the minor issue for this manuscript

**Were you able to directly test the methods?**

No

**Comments to author:**

Przytycki et al describe CellWalker, a method that integrates scATAC-seq data with RNA-seq and other data types that can simultaneously improves cell labeling in noisy scATAC-seq and annotates cell-type specific regulatory elements in bulk data. The authors then applied CellWalker to the developing brain, and claimed they had identified cells transitioning between transcriptional states, resolved enhancers to specific cell types, and observed that autism and other neurological traits can be mapped to specific cell types through their enhancers. The manuscript is badly written and the method is very obscure. Overall this work is very low quality and lacks importance, quantitative measurements and rigorous comparison, and thereby has not reached the point where it can be published in Genome Biology.

Specific comments are as follows:

1. The results in the manuscript are not sufficient to prove the conclusion. The authors claimed that CellWalker "simultaneously improves cell labeling in noise scATAC-seq and annotates cell-type specific regulatory elements in bulk data". To what extend CellWalker "improves" cell labeling? How much better it compares to other state-of-the-art scATAC-seq analysis algorithms?

2. The description of the method is very unclear, which makes it extremely difficult for readers to understand what exactly CellWalker is doing. What is the logic of the model and how can a potential user benefit from CellWalker? Why CellWalker performs better (if it does) than other methods? How to define similarity? What is cell homogeneity? Do edge weights need to be optimized or it is integrated in CellWalker?

3. There are quite a few scATAC-seq analysis algorithms, such as snapATAC, APEC, cisTopic, LSI, Cicero and etc. How does the performance of CellWalker compare to these methods? There is hardly any comparison in the manuscript.

4. The language of the whole article is obscure, and I can hardly understand what the authors want to explain.

There are so many issues that I can easily found unclear, I just give a few examples:

1. "CellWalker achieves this efficiently in terms of both time and computer memory usage". This is based on 10,000 cells, how about the performance of CellWalker processing 100,000 or millions of cells?

2. In other systems such as PBMC or HSC development, is the CellWalker still performs better than Garnett and Seurat?

3. How sensitive does the single label edge weight parameter to different data? How fast it is to compute cell homogeneity.

4. Line 189: "This appears to be a biological difference between the dynamics of gene expression versus open chromatin during developmental transitions, though higher coverage scATAC-seq data could potentially alter this conclusion", is there evidence to prove this?

5. Line 201: "found that the distribution of correlations is significantly bimodal (empirical p-value = 0.021)", No results of TAD were found.

6. Line 219: "Notably, the neurogenic differentiation gene NEUROD1 lies in a late TAD with higher levels of accessibility late than early throughout the TAD but similar accessibility in the gene body and promoter". There is no Figure 3D, there is only Figure 3c in figure. And I did not find the result of TENM4.

7. Can CellWalker uncover cell-type specific TFs based on a combination of bulk and single cell data?

8. The tutorial is too simple, and some of the functions mentioned in the article are not shown in the tutorial, such as TAD, enhancer and disease genes. The example data and reference data are not provided, the author should provide a download link for users to download and use.

9. The authors claim "we observe cells that score highly as combinations of intermediate progenitor cells, early newborn excitatory neurons, late newborn excitatory neurons, and maturing excitatory neurons (Figure 3b, group 3), suggesting that these transcriptional states cannot be resolved in scATAC-seq." is there evidence to prove this?

10. In Fig 3, the authors should at least compare the performance of CellWalker with SnapATAC to prove that CellWalker is better than SnapATAC in identifying rare cell types.

11. In section of "Cell-type specific annotation of loci.", the authors use Hi-C data to say that "the accessibility of GZ TADs distinctly either correlates or anti-correlates with cell state progression from early to late excitatory neuron." Without convincing evidence. Supp Fig. 4b&c is absolutely far from enough.

12. What does this sentence mean? "Nonetheless, we observed that most cell belong strongly to one type, indicating that most transcriptional states observed in scRNA-seq are associated with a distinct open chromatin signature in scATAC-seq"

**Authors Response**

**Point-by-point responses to the reviewers’ comments:**

Reviewer #1

1) The method section on how the algorithm work needs more details. I found the information that the authors provided in the GitHub are quite helpful. I'd suggested that the authors include those GitHub contents to this manuscript and provide a flowchart to better demonstrate the overall workflow of CellWalker.

*Thank you for the great suggestion, we have added a flowchart describing CellWalker to the paper (Supplemental Figure 1). We have also rewritten the methods section into smaller discrete steps more in-line with the flow of the GitHub page.*

2) The authors used the ATAC-seq signals around promoters and gene bodies to link the gene-expression based label information to cells. It seems that the distal enhancers, which are more important to tissue-specific expression, were not included when building these label-cell edges. Can the authors explain the rationale behind?

*We agree that a distal-enhancer focused analysis would be interesting. Our method can take as input any quantifications, including ones like CICERO that consider enhancers. In our manuscript we decided to use the SnapATAC pipeline throughout and hence followed their guideline to work with promoters and gene bodies. We have updated our manuscript to mention this decision and to explain the upstream choices that can be made prior to applying CellWalker. We also analyzed predicted enhancers from Markenscoff-Papadimitriou et al., many of which are distal regulators.*

3) scATAC-seq in human telencephalon is one of the major data set the authors used to demonstrate the power of CellWalker. However, description of this data set is not clear. I can understand that the authors tried to avoid introducing overlapping contents with another manuscript which seems still under review. However, there should be enough information provided so that the readers do not have to read through another manuscript just for some fundamental information. For examples, How the cells for scATAC-seq were prepared in that study? How CellWalker improved the annotation of different cell populations, especially compared with other approaches?

*Thanks for pointing this out. We agree that clear descriptions of data are critical. Our manuscript describing the telencephalon data and its processing in detail has now been accepted (Ziffra et al., Nature). We have updated the description of the data in this manuscript to provide additional details relevant to its analysis with CellWalker, with a particular focus on what CellWalker improves. We refer readers to Ziffra et al. for other details. Of note, relative to other methods that were tested on the same data in the Ziffra et al. paper, only CellWalker was able to distinguish between different types of radial glia, and to separate the two major types of MGE-derived inhibitory interneurons.*

4) In "Cell-type specific annotation of loci" section. Instead of directly jumping to the aggregated analysis of signals within TADs, what is the genome-wide accessibility change of individual ATAC-seq peak among different cell types annotated by CellWalker? It seems some pieces information were missing in this section. In addition, it is not accurate to call those cell-type specific TADs, since I did not find any data showing that the TAD boundaries changed among different cell types.

*We avoided doing genome-wide cell-type peak accessibility analysis for this manuscript since that would overlap with results from the cited Ziffra et al. paper. However we have added a few relevant analyses to this manuscript, such as noting that 92% of early nEN peaks are also found in late nEN to show how similar the two types are.*

*Thank you for pointing out this inaccuracy in our description of cell-type specific TADs. What we actually mean is whether a given TAD is active in a cell type, not whether the TAD itself is different. In the revision, we no longer call these cell-type specific TADs, and we clarify the analysis.*

5) In "Cell-type specific annotation of enhancers" section. Again, due to the limited information on data sets description. It is difficult to judge the biological relevant of the bulk ATAC-seq and scATAC-seq data used in the analysis. In addition, I do not feel the author have enough information to call those ATAC-seq peaks enhancers. "Regulatory elements" is more accurate.

*We agree and have changed our naming throughout to refer to these as “predicted regulatory elements (pREs)” as they are called in the Markenscoff-Papadimitriou et al. paper from which they were taken. We have also updated the text to clarify that these are regions predicted to be regulatory using machine learning trained on bulk epigenetic data and vista enhancers.*

Minor points.

1) Line 121 "When edge weight is low, the output is similar to de- novo cell clustering using only scATAC-seq" one comma is missing between the two sentences.

*Thank you, this has been corrected.*

2) Line 159 : only the cluster of scATAC-seq data is shown in Figure 3a, I failed to see " high agreement with cluster based on scRNA-data"

*What we were hoping to get across is that our clustering reflects the scRNA-seq clustering from the previously published Nowakowski et. al paper which we did not reproduce in our text. We have clarified this as well as added a description of similarities including broad agreement such as that all radial glia cell types group together, as well as local similarities such as nEN-early1 being more similar to EN-PFC3 than other newborn excitatory neuron types.*

3) Line 202, pls show the distribution instead of only a p-value.

*We have added a plot of the full distribution as well as the distribution of correlations for randomized TADs to the supplement (reproduced in response pdf).*

Reviewer #2

1) The results in the manuscript are not sufficient to prove the conclusion. The authors claimed that CellWalker "simultaneously improves cell labeling in noise scATAC-seq and annotates cell-type specific regulatory elements in bulk data". To what extend CellWalker "improves" cell labeling? How much better it compares to other state-of-the-art scATAC-seq analysis algorithms?

*We believe we have shown CellWalker to improve cell labeling in noisy data via several avenues. First, with extensive simulation data we found that CellWalker could label cells more accurately than labeling based on cell clustering or than directly applying labels to data. Then, in order to evaluate CellWalker on real data we used SNARE-seq data which generates scRNA scATAC-seq data for each cell. We compared Seurat to CellWalker on this data and found CellWalker to more accurately label cells in scATAC-seq. We have now added evaluations for a second set of SNARE-seq data.*

*Most existing scATAC-seq methods are not intended to solve the same problem of cell labeling that Seurat and CellWalker address (e.g. they may map scRNA-seq and scATAC-seq data into the same space or call peaks in scATAC-seq clusters). Because these methods do not produce cell type labels, a direct comparison is not feasible. We did explore extending one of them, Garnett, to produce labels and perform an evaluation. However, Garnett’s performance was quite poor, likely because it was not designed to solve this problem. Hence, we did not feel it would be fair to include a comparison.*

*The reviewer’s comments raise an important point. There are many models and bioinformatics pipelines for scATAC-seq data. To understand why a new method is needed and if it offers value to scientists, it is critical to clarify what problems the different tools solve and where there is room for new methods development. We did not do this well in the initial submission, and we thank the reviewer for pointing this out. In our revision, we attempt to clearly describe the range of currently available tools, what they each do, and which ones are comparable to CellWalker versus solving a different problem upstream or downstream of our method.*

2) The description of the method is very unclear, which makes it extremely difficult for readers to understand what exactly CellWalker is doing. What is the logic of the model and how can a potential user benefit from CellWalker? Why CellWalker performs better (if it does) than other methods? How to define similarity? What is cell homogeneity? Do edge weights need to be optimized or it is integrated in CellWalker?

*Providing a clear problem statement and defining the terms we use to evaluate performance are critical. To improve both of these points, we have made major changes and additions to the manuscript. First, we added a flowchart to the manuscript in order to make CellWalker’s analysis pipeline clearer. Additionally, we have extensively rewritten for clarity including delineating how CellWalker relates to other methods (e.g. they can be used as input,or they can be used to process output). One element made more clear in the flowchart and text is that similarity can be defined flexibly by the user. In our analysis we chose to use Jaccard similarity. Cell homogeneity, as defined in the methods, is the median ratio of information between cells within the same cell type to information between cells of different cell types. Finally, edges have two stages at which their weights can be set (made more clear in the flowchart). In the initial edge creation step, starting values for weights can be generated based on the output of a program such as SnapATAC or Cicero, or they can be calculated using gene-body or promoter peaks. These weights are then optionally optimized internally by CellWalker to maximize cell homogeneity.*

3) There are quite a few scATAC-seq analysis algorithms, such as snapATAC, APEC, cisTopic, LSI, Cicero and etc. How does the performance of CellWalker compare to these methods? There is hardly any comparison in the manuscript.

*These methods generally function upstream or downstream of CellWalker. We have clarified this in the text, and in the new flowchart figure we note where each method could be applied. For example, SnapATAC and CICERO make activity matrices that can be used as input to CellWalker, while cisTopic can be run on CellWalker’s output.*

4) The language of the whole article is obscure, and I can hardly understand what the authors want to explain.

*We appreciate the feedback that our manuscript should be easy for diverse readers to understand. To address this point, we have rewritten the manuscript to make it clearer, with a particular focus on the methods sections. We split long sections into subsections with one idea each. We also added some topic sentences to each section emphasizing the big picture ideas and how the results could help a reader. Finally, we have had a professional writer edit the text.*

Other Points:

1) "CellWalker achieves this efficiently in terms of both time and computer memory usage". This is based on 10,000 cells, how about the performance of CellWalker processing 100,000 or millions of cells?

*This question about scaling is important as scATAC-seq data sets continue to grow in terms of the number of sequenced cells. We highlighted results for 10,000 cells as currently most scATAC-seq experiments sequence up to that many. Looking ahead, we set out to test the upper limits of CellWalker and found that 50,000 cells would still be feasible to analyze on a powerful compute node (11.7 hours and 788Gb of RAM) while 100,000 cells would require a high-performance cluster (~80 hours and >3 Tb of RAM). These results have been added to the manuscript.*

2) In other systems such as PBMC or HSC development, is the CellWalker still performs better than Garnett and Seurat?

*In order to assess the performance of CellWalker and related methods, data with known cell labels is required, in addition to any data that other methods need. Thus we evaluated CellWalker on SNARE-seq experiments which have all the necessary data. We note that we attempted a comparison to Garnett by extending its functionality. With the given data (matched RNA-seq), Garnett was unable to label more than 50% of cells. Thus we deemed it would be unfair to compare to CellWalker, since Garnett was not designed with scATAC-seq labeling in mind. However, as a less exact comparison of performance, we ran Seurat on PBMC data to generate a hierarchical clustering of cell types (see figure in response pdf). We compared this to the cell type clustering from CellWalker. While there are some differences, both generated reasonable and similar hierarchies, with the only notable difference being the placement of NK cells. Without a gold standard, as in SNARE-seq or a simulation, the best we can do is to say that Seurat and CellWalker give similar results.*

3) How sensitive does the single label edge weight parameter to different data? How fast it is to compute cell homogeneity.

*The edge weight parameter is different for each data set and can vary by orders of magnitude. We have added a point to our discussion section to comment on the meaning of this parameter. Computing cell homogeneity is very fast (we improved code efficiency since the initial submission) taking less than a second per cell type with 10,000 cells, and even less time with 5,000 cells (see figure in response pdf). These results have been added to the manuscript.*

4) Line 189: "This appears to be a biological difference between the dynamics of gene expression versus open chromatin during developmental transitions, though higher coverage scATAC-seq data could potentially alter this conclusion", is there evidence to prove this?

*We have dropped the word biological from this section as we don’t have a validation experiment. Rather we meant to say we observed a difference that may not hold up with higher coverage data.*

5) Line 201: "found that the distribution of correlations is significantly bimodal (empirical p-value = 0.021)", No results of TAD were found.

*We have added a plot of the distribution as well as a plot of correlations for random TADs as compared to real TADs to the supplement (reproduced with minor point 3 from Review #1 above).*

6) Line 219: "Notably, the neurogenic differentiation gene NEUROD1 lies in a late TAD with higher levels of accessibility late than early throughout the TAD but similar accessibility in the gene body and promoter". There is no Figure 3D, there is only Figure 3c in figure. And I did not find the result of TENM4.

*Thank you for catching this. We have corrected the text to read 3c rather than 3d. We did not include a figure for TENM4 as we thought it might be redundant to show both genes, visually the panels would look very similar. TENM4 is now shown in the supplement.*

7) Can CellWalker uncover cell-type specific TFs based on a combination of bulk and single cell data?

*This is an interesting idea. We tested if this was feasible using Cortex, GE, and V1/PFC specific TFs combined with bulk data from Markenscoff-Papadimitriou et. al. Using HOMER we found all bulk peaks that each TF was predicted to bind to, and then mapped these peaks to cell types using the cell-to-label information from CellWalker. We observed that all the cortex-specific TFs mapped to IPC-nENs (IPC-nEN1 or IPC-nEN2), all the GE-specific TFs mapped to MGE-IPCs, and V1/PFC specific TFs all mapped to EN-PFC3 (with the exception of one that mapped to IPC-nEN1). We also found that TFs with highly similar motifs map to the same cell types, as expected. While these mappings are entirely plausible (the cell types are all of the correct broad type), it is hard to say if they are specific (i.e., the TFs may also map to other cell types). We hypothesize that this result is driven in part by the fact that these were fairly dominant cell types within the bulk samples from Markenscoff-Papadimitriou et. al. Thus we can establish these TFs as positives for these cell types, but we cannot be sure about false negatives. We have added this functionality to our GitHub as the results are interesting even given the propensity for false negatives.*

8) The tutorial is too simple, and some of the functions mentioned in the article are not shown in the tutorial, such as TAD, enhancer and disease genes. The example data and reference data are not provided, the author should provide a download link for users to download and use.

*Thanks for this good suggestion. We have expanded the tutorial on GitHub to include downstream analysis as well. We have added specific descriptions for where data can be found and which papers it was taken from, though we note that some is not publicly available yet.*

9) The authors claim "we observe cells that score highly as combinations of intermediate progenitor cells, early newborn excitatory neurons, late newborn excitatory neurons, and maturing excitatory neurons (Figure 3b, group 3), suggesting that these transcriptional states cannot be resolved in scATAC-seq." is there evidence to prove this?

*For clarity, we have rephrased this to: Some transcriptional states are difficult to distinguish from each other. For example, some cells score highly as two or more of the following types: intermediate progenitor cells, early newborn excitatory neurons, late newborn excitatory neurons, and maturing excitatory neurons.*

10) In Fig 3, the authors should at least compare the performance of CellWalker with SnapATAC to prove that CellWalker is better than SnapATAC in identifying rare cell types.

*We note that SnapATAC does not have a method to identify cell types, rather it clusters them and then assigns cell types using Seurat. CellWalker is an alternative to the Seurat step, and like Seurat can use SnapATAC (or other quantification methods) as input. As such, both methods resolve cell types from SnapATAC output. In terms of comparison, the Ziffra et al. paper manually assigned cell types to clusters generated by SnapATAC and also compares these to CellWalker resolved cell types. That analysis shows that CellWalker is able to assign cell labels at a higher resolution, identifying cells from every cell type in Nowakowski et al. Notably, we were able to distinguish between different types of radial glia as well as between different types of cortical derived interneurons using CellWalker. We now refer the reader to these results in the other publication.*

11) In section of "Cell-type specific annotation of loci.", the authors use Hi-C data to say that "the accessibility of GZ TADs distinctly either correlates or anti-correlates with cell state progression from early to late excitatory neuron." Without convincing evidence. Supp Fig. 4b&c is absolutely far from enough.

*We explicitly tested the null hypothesis that the correlation distribution is no more biomdal than expected by chance and found statistical evidence for bimodality. The full distribution is now included in the supplement. Supplemental figures 4b & c (now supplemental figures 5 d & e) are controls for other possible covariates.*

12) What does this sentence mean? "Nonetheless, we observed that most cell belong strongly to one type, indicating that most transcriptional states observed in scRNA-seq are associated with a distinct open chromatin signature in scATAC-seq"

*For most cells in the scATAC-seq (27,270 out of 30,000) we were able to assign a label from transcriptional states (scRNA-seq) without much ambiguity. This result was not described as clearly as it could have been. We have rephrased this finding in the paper and added more details to clarify figure 3b.*

**Second round of review**

**Reviewer 1**

The authors have addressed my main concerns.

The order of some contents in the revised manuscript needs to be adjusted. For example, the concept of "Cell Homogeneity" in Figure 2a (Line 120) is introduced in line 129. Similar, the abbreviation of cell types in figure 3a should be mentioned earlier than the current version. I suggest the authors carefully work on these details to improve the clarify.

**Reviewer 2**

The revised manuscript is way much better and clearer. All my previous comments have been addressed.
